# The fatty acid-related gene signature stratifies poor prognosis patients and characterizes TIME in cutaneous melanoma

**DOI:** 10.1007/s00432-023-05580-7

**Published:** 2024-01-27

**Authors:** Shan Hua, Wenhao Wang, Zuochao Yao, Jiawei Gu, Hongyi Zhang, Jie Zhu, Zhiwen Xie, Hua Jiang

**Affiliations:** 1 Department of Plastic Surgery, Shanghai East Hospital, Tongji University School of Medicine, 150 Jimo Road, Shanghai, 200120 China; 2 Department of Urology, Shanghai General Hospital, Shanghai Jiao Tong University School of Medicine, Shanghai, China

**Keywords:** Cutaneous melanoma, Fatty acid, Immune microenvironment, Prognostic signature, Nomogram, Immunotherapy

## Abstract

**Background:**

The aim of this study is to build a prognostic model for cutaneous melanoma (CM) using fatty acid-related genes and evaluate its capacity for predicting prognosis, identifying the tumor immune microenvironment (TIME) composition, and assessing drug sensitivity.

**Methods:**

Through the analysis of transcriptional data from TCGA-SKCM and GTEx datasets, we screened for differentially expressed fatty acids-related genes (DEFAGs). Additionally, we employed clinical data from TCGA-SKCM and GSE65904 to identify genes associated with prognosis. Subsequently, utilizing all the identified prognosis-related fatty acid genes, we performed unsupervised clustering analysis using the ConsensusClusterPlus R package. We further validated the significant differences between subtypes through survival analysis and pathway analysis. To predict prognosis, we developed a LASSO-Cox prognostic signature. This signature's predictive ability was rigorously examined through multivariant Cox regression, survival analysis, and ROC curve analysis. Following this, we constructed a nomogram based on the aforementioned signature and evaluated its accuracy and clinical utility using calibration curves, cumulative hazard rates, and decision curve analysis. Using this signature, we stratified all cases into high- and low-risk groups and compared the differences in immune characteristics and drug treatment responsiveness between these two subgroups. Additionally, in this study, we provided preliminary confirmation of the pivotal role of CD1D in the TIME of CM. We analyzed its expression across various immune cell types and its correlation with intercellular communication using single-cell data from the GSE139249 dataset.

**Results:**

In this study, a total of 84 DEFAGs were identified, among which 18 were associated with prognosis. Utilizing these 18 prognosis-related genes, all cases were categorized into three subtypes. Significant differences were observed between subtypes in terms of survival outcomes, the expression of the 18 DEFAGs, immune cell proportions, and enriched pathways. A LASSO-Cox regression analysis was performed on these 18 genes, leading to the development of a signature comprising 6 DEFAGs. Risk scores were calculated for all cases, dividing them into high-risk and low-risk groups. High-risk patients exhibited significantly poorer prognosis than low-risk patients, both in the training group (*p* < 0.001) and the test group (*p* = 0.002). Multivariate Cox regression analysis indicated that this signature could independently predict outcomes [HR = 2.03 (1.69–2.45), *p* < 0.001]. The area under the ROC curve for the training and test groups was 0.715 and 0.661, respectively. Combining risk scores with clinical factors including metastatic status and patient age, a nomogram was constructed, which demonstrated significant predictive power for 3  and 5 years patient outcomes. Furthermore, the high and low-risk subgroups displayed differences in the composition of various immune cells, including M1 macrophages, M0 macrophages, and CD8^+^ T cells. The low-risk subgroup exhibited higher StromalScore, ImmuneScore, and ESTIMATEScore (*p* < 0.001) and demonstrated better responsiveness to immune therapy for patients with PD1-positive and CTLA4-negative or positive expressions (*p* < 0.001). The signature gene CD1D was found to be mainly expressed in monocytes/macrophages and dendritic cells within the TIME. Through intercellular communication analysis, it was observed that cases with high CD1D expression exhibited significantly enhanced signal transductions from other immune cells to monocytes/macrophages, particularly the (HLA-A/B/C/E/F)-CD8A signaling from natural killer (NK) cells to monocytes/macrophages (*p* < 0.01).

**Conclusions:**

The prognostic signature constructed in this study, based on six fatty acid-related genes, exhibits strong capabilities in predicting patient outcomes, identifying the TIME, and assessing drug sensitivity. This signature can aid in patient risk stratification and provide guidance for clinical treatment strategies. Additionally, our research highlights the crucial role of CD1D in the CM's TIME, laying a theoretical foundation for future related studies.

**Supplementary Information:**

The online version contains supplementary material available at 10.1007/s00432-023-05580-7.

## Introduction

Cutaneous melanoma (CM) is the third most commonly diagnosed fetal skin cancer, following basal cell carcinoma and squamous cell carcinoma. The incidence of CM continues to increase, while that of many other cancers is decreasing (Miller and Mihm 2006; MacKie et al. 2009). According to GLOBOCAN 2020 data, there were over 300,000 diagnoses and 60,000 deaths worldwide in 2020 (Sung et al. 2021). The mortality rate associated with CM, with highly metastatic nature, surpasses that of non-melanoma cancers by fourfold (Arheden et al. 2019). Even though CM patients initially present with localized disease, potentially curable through primary tumor resection, a significant number proceed to develop distant metastases, leading to a dire prognosis (Rastrelli et al. 2014). Advanced tumor stage and unfavorable prognosis are intricately linked to delays in diagnosis. The absence of patient recognition regarding early-stage CM’s clinical characteristics, combined with a lack of awareness and knowledge concerning the disease, contribute to this outcome (Klebanov et al. 2019). Therefore, high cure rate in early stage and high mortality in advanced stage mean that early-stage diagnoses and predicting therapeutic responsiveness and prognosis for patients are of significant clinical importance. Consequently, the marked contrast between high cure rates in the early stage and elevated mortality in the advanced stage underscores the pivotal clinical significance of early-stage diagnoses and predictions for therapeutic responsiveness and patient prognosis.

Fatty acids (FAs) are a diverse group of molecules consisting of hydrocarbon chains that vary in length and saturation levels. They are essential components of lipids, which are energy-dense compounds. When metabolized, lipids produce a significant amount of ATP, necessary for various cellular activities. As a result, the survival of tumor cells is largely influenced by processes such as lipid synthesis, metabolism, and degradation (Shevchenko and Simons 2010). On one hand, tumor cells can acquire FAs via de novo biosynthesis (Ookhtens et al. 1984). However, in scenarios where endogenous FAs are deficient, like when the entry of pyruvate into the tricarboxylic acid cycle is halted due to hypoxia, tumor cells can absorb exogenous FAs from the tumor microenvironment (TME) (Papandreou et al. 2006; Kamphorst et al. 2013). Multiple studies have emphasized the crucial role FAs play in the proliferation and sustenance of various cancer cells (Santos and Schulze 2012). Within cancer cells, the reactivation of FA synthesis may indicate a regression of the tissue to a poorly differentiated embryonic state or an adaptation to the TIME's characteristic low serum lipid content (Kusakabe et al. 2000). Besides, FAs are also associated with tumor progression and drug resistance. The selective uptake or release of specific FAs from membrane lipids in tumor tissues can facilitate the synthesis of signaling molecules, thereby promoting migration and invasion (Nath et al. 2015). Epithelial-mesenchymal transition processes also require lipid remodeling to modify membrane fluidity essential for cell migration (Zhao et al. 2016). Consequently, lipid metabolism, with a particular focus on FA metabolism, is increasingly viewed as a promising therapeutic target for cancers. For CM cells, glycolysis and oxidative metabolism are primary energy sources, with the latter being predominant. An elevated FA content, vital for oxidative metabolism, is associated with drug resistance and metastasis in melanoma (Fischer et al. 2019). Recent researches suggest that CM cells undergo fatty acid oxidation (FAO) either using existing lipid reserves or by uptaking exogenous lipids through fatty acid transporters. Melanocytic cells store FAs within lipid droplets, and disrupting these droplets can impair the function of FAs, leading to cell cycle interruption and hindered CM progression (Lumaquin-Yin et al. 2023; Shin et al. 2023). Hence, targeting FA metabolism emerges as a promising therapeutic strategy for treating CM.

Historically, chemotherapy has been a mainstay in CM treatment. However, recent advancements in immunotherapy, especially with the introduction of immune checkpoint inhibitors (ICIs) and BRAF-MEK inhibitors, have shown significant anti-tumor activity and therapeutic benefits (Ma et al. 2023). Immunotherapy has notably improved the outcomes for CM patients. Data suggest that the 5 years survival rate for stage IV patients treated with a single-agent PD1 inhibitor ranges from 34 to 44%. Impressively, this rate escalates to 52% when supplemented with the combination CTLA4 inhibitor, ipilimumab (Pham et al. 2023). However, the therapeutic landscape isn’t without challenges. Resistance to immunotherapy, often stemming from individual genetic and epigenetic factors, can limit the treatment's efficacy. Such resistance can be attributed to complex signaling interactions between cancer cells and the TME, varying across individuals (Indini et al. 2023). Reports indicate that the metabolic interplay of FAs within the TIME can influence the content and functionality of a diverse set of immune cells (Koundouros et al. 2020). For instance, the accumulation of FAs in Natural Killer (NK) cells inhibits the production of IFN-γ and cytotoxic granules, thereby suppressing their anti-tumor capabilities (Michelet et al. 2018). Tumor-associated conventional dendritic cells (cDCs), exhibiting heightened MSR1 expression, are prone to substantial FA uptake. Such abnormal FA accumulation can impair their antigen presentation capabilities (Herber et al. 2010). Moreover, tumor-associated macrophages (TAMs) adjust to the TIME, in part through FA metabolic reprogramming. A high expression of CD36 in TAMs augments FA oxidation and oxidative phosphorylation, polarizing TAMs towards M2-like phenotypes (Su et al. 2020).

FAs are instrumental in the onset, progression, treatment, and immune microenvironment of CM. Despite their significance, no prognostic signature based on fatty acid-related genes currently exists. In this study, our objective is to establish a LASSO-Cox prognostic signature by utilizing gene expression profiles and clinical data from public databases, and to assess its predictive capacity in terms of prognosis, immune characteristics, and immunotherapeutic responsiveness among CM patients.

## Materials and methods

### Raw data acquisition

RNA sequencing (RNA-seq) profiles and clinical data of SKCM (Skin Cutaneous Melanoma) were obtained from the TCGA-SKCM dataset in The Cancer Genome Atlas (TCGA) (https://tcga-data.nci.nih.gov/tcga/) and the GSE65904 dataset in the Gene Expression Omnibus (GEO) database (https://www.ncbi.nlm.nih.gov/geo/). Since the TCGA-SKCM dataset had only one normal skin sample available, additional transcript data were sourced from the Genotype-Tissue Expression (GTEx) database (https://commonfund.nih.gov/GTEx) to facilitate the identification of differentially expressed genes. Fatty acid-related genes were obtained from various datasets, including Hallmark, Reactome, KEGG, and WP datasets in GSEA Human MSigDB v2023.1.Hs (https://www.gsea-msigdb.org/gsea/msigdb/index.jsp). Additionally, the single-cell RNA sequencing (scRNA-seq) dataset GSE139249 for melanoma was acquired from the GEO database.

### Screening differentially expressed fatty acid-related genes (DEFAGs)

RNA-seq profiles from TCGA-SKCM and GTEx were removed the batch effects with sva R packages. Differentially expressed genes (DEGs) between normal skin samples and melanoma samples were screened out with limma R package. Statistically significant DEGs were defined using |LogFC|> 1 and fdr < 0.05 as cut-offs. Genes in the intersection between DEGs and fatty acid gene list were regarded as DEFAGs used for further analysis.

### Unsupervised cluster analysis

Using the overall survival data from the TCGA-SKCM and GSE65904 datasets, survival analysis was performed for each DEFAG using the survival and survminer R packages. Based on the identified 18 prognosis-related genes, all SKCM samples were categorized into different subtypes using the ConsensusClusterPlus R package. The most appropriate number of subtypes was determined through the consensus clustering algorithm. To evaluate the results of this clustering, we employed Principal Component Analysis (PCA) and t-Distributed Stochastic Neighbor Embedding (tSNE) methods.

### Pathway enrichment analysis

The infiltration of immune cells in each subtype was assessed using the single-sample gene set enrichment analysis (ssGSEA) algorithm, facilitated by the clusterProfiler and org.Hs.eg.db R packages. Additionally, Gene Set Variation Analysis (GSVA) and Gene Set Enrichment Analysis (GSEA) were employed to identify disparities in enrichment pathways across different subtypes. These analyses were conducted using the GSVA and GSEABase R packages.

### Construction and evaluation of prognostic signature

Prognosis-related DEFAGs underwent processing using the LASSO method to mitigate overfitting and to exclude closely related genes. The optimal penalty parameter (*λ*) was determined via fivefold cross-validation. Subsequently, a LASSO-Cox signature for SKCM was formulated. Using this signature, all melanoma samples were categorized into either high-risk or low-risk subgroups. The assignment was based on risk scores calculated using the given formula, with the median serving as the cut-off point. The prognostic performance of the signature was evaluated using Receiver Operating Characteristic (ROC) analysis, facilitated by the timeROC R package. Furthermore, multivariate Cox regression was employed to determine if the signature functions as an independent prognostic factor.

### Construction of nomogram

To further quantitatively assess the signature, we developed a comprehensive nomogram utilizing independent factors for SKCM with the rma R package. Calibration curves for 1, 3, and 5 years timeframes, as well as cumulative hazard curves, were utilized to evaluate the accuracy of the nomogram. Additionally, the clinical utility of the nomogram was evaluated using decision curve analysis (DCA).

### Comprehensive analysis of immune characteristics in different subgroups

The expression matrix of all SKCM samples sourced from TCGA-SCKM and GSE65904 was submitted to the CIBERSORT database for the analysis of immune cell proportions across each melanoma sample. A comprehensive landscape map was generated, detailing the outcomes in a bar plot. Moreover, differing infiltration levels of immune cells within the high- and low-risk subgroups were visualized using a violin plot. Furthermore, the correlation between the infiltration levels of these immune cells and each candidate gene within this signature—as well as risk scores—was ascertained and visualized in a heatmap. The tumor purity across different subgroups was estimated and compared using the Estimation of Stromal and Immune cells in Malignant Tumor tissues using Expression data (ESTIMATE) method.

### Prediction of immunotherapy response and chemosensitivity

The immunophenoscore (IPS) can reflect the efficacy of immune checkpoint inhibitors (Charoentong et al. 2017). We retrieved IPS for samples in TCGA from the Cancer Immunome Atlas (TCIA, https://tcia.at/home) and compared the immunotherapy response between different subgroups. The chemosensitivity of each sample in TCGA and GSE65904 was estimated with oncoPredict R package and compared between subgroups with Wilcox t test.

### Single cell RNA sequencing analysis

Initially, the correlation between each gene within this signature and immune cell infiltration levels was examined using TIMER 2.0 (http://timer.cistrome.org/) to identify the gene with the most pronounced influence on the tumor’s immune environment. Subsequently, the scRNA-seq dataset (GSE139249) for melanoma was retrieved from the GEO database. Quality control and normalization were carried out using DropletUtils and the Seurat R package, respectively. Cell population clustering was performed using the findNeighbors and findClusters functions in Seurat, with the UMAP (Uniform Manifold Approximation and Projection) method employed to visualize cell clusters. The analysis of intercellular communication was conducted using the CellChat R package (Jin et al. 2021).

## Results

### Fatty acid-related hub genes

**Fig. 1:**
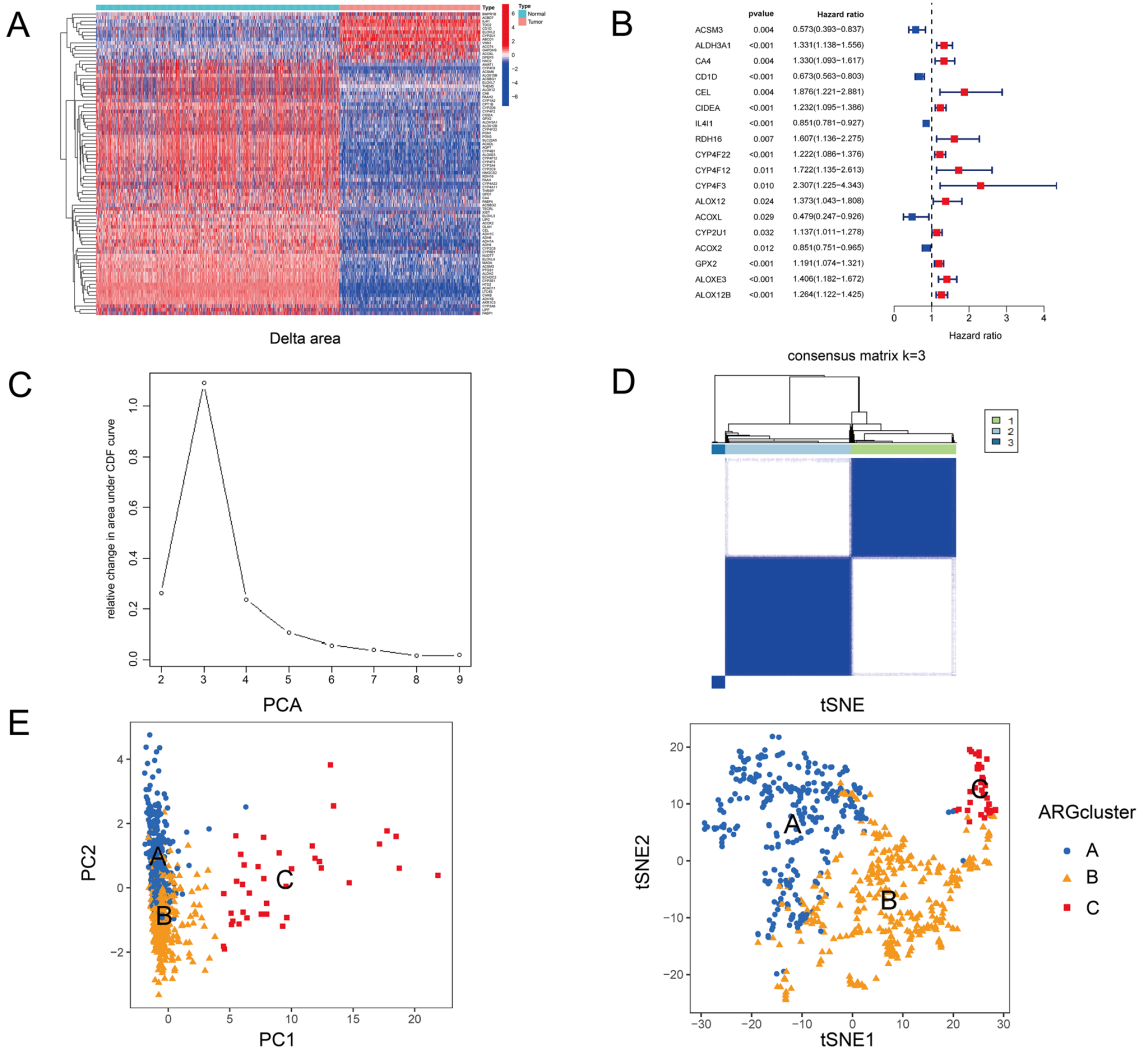
**A** Heatmap shows 13 DEFAGs upregulated and 71 DEFAGs downregulated in tumor samples. **B** Forest plot shows 18 DEFAGs are related to patients’ prognosis. **C**–**D** Delta area plot indicates the optimal *K* value as 3, and when *K* = 3, the consensus matrix heatmap exhibits the cleanest cluster partition. Based on this, all melanoma patients are separated into three subtypes. **E** Both PCA and tSNE methods shows significant heterogeneity among three subtypes

The transcript data of a total of 812 normal skin samples were collected from GTEx, 1 normal and 471 melanoma samples were collected from TCGA, and 44 melanoma samples were collected from GSE19234. Clinicopathological and prognostic data were retrieved for 44 cases in GSE19234 and 412 cases in TCGA (Table 1). A list of 339 fatty acid-related genes is provided in Table S1. After removing batch effect and conducting analysis of variance, 84 DEFAGs were identified. Among these, 13 genes exhibited up-regulation while 71 genes displayed down-regulation in tumor samples (Table S2, Fig. 1A). Subsequently, survival analysis was performed based on their expression profiles and survival data, leading to the identification of 18 DEFAGs that significantly affect prognosis for melanoma patients. Among the 18 genes, IL4I1A, ACOXL, and CYP2U1 are upregulated in CM, while ACSM3, ALDH3A1, CA4, CD1D, CEL, CIDEA, RDH16, CYP4F22, CYP4F12, CYP4F3, ALOX12, ACOX2, GPX2, ALOXE3, and ALOX12B are downregulated. These genes were then used for subsequent clustering analysis and signature construction (Fig. 1B).

**Table 1:** Sources of raw data for normal and CM RNA-seq data and CM’S clinical data

Data type	GTEx	TCGA-SKCM	GSE19234	Total
RNA-seq of normal samples, *N*	812	1	0	813
RNA-seq of Tumor, *N*	0	471	44	515
Clinical data, *N*	0	412	44	456

### Cluster construction and characteristics comparison

**Fig. 2:**
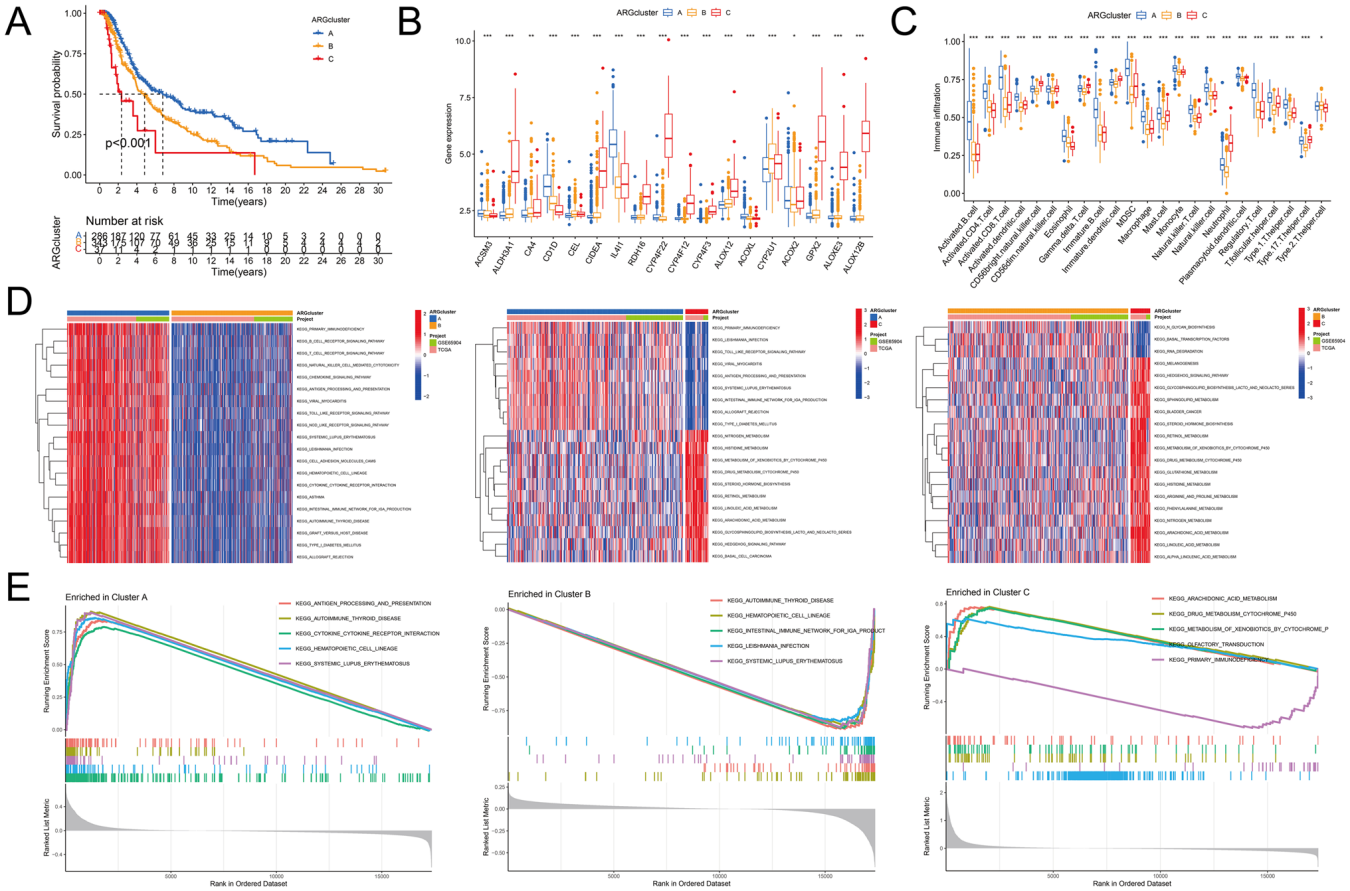
**A** Survival analysis demonstrates a significant difference in prognosis among the three subtypes (*p* < 0.001). **B** The expression levels of 18 DEFAGs show significant differences among the three subtypes. **C** ssGSEA results shows the proportions of 23 immune cells are different among the three subtypes. **D** GSVA results indicate significant differences in enriched pathways among the three subtypes. **E** GSEA results reveal the top five enriched pathways for each of the three subtypes. *p < 0.05, **p < 0.01, ***p < 0.001

Consensus clustering is an unsupervised clustering method that categorizes specific cancers into subtypes based on DEFAGs expression patterns. It is used for the identification of new subtypes and for conducting comparative analysis between different subtypes. The Delta area plot exhibits an inflection point at *x* = 3, and when *k* = 3, the consensus matrix heatmap displays the cleanest cluster partition (Fig. 1C–D). Based on the delta area plot and the distinct boundaries of subtypes in consensus matrix heatmap, the melanoma patients were separated into three subtypes. Two dimensionality reduction methods (PCA and tSNE) highlighted significant heterogeneity among three subtypes, thus confirming the reliability and success of our clustering (Fig. 1E). Results from survival analysis demonstrated substantial survival differences among the three subtypes (*p* < 0.001) (Fig. 2A). This to some extent implies that the differing expression patterns of DEFAGs can impact the prognosis of CM patients. Expression differences of the 18 DEFAGs across the subtypes were visualized in Fig. 2B. The ssGSEA results indicated varying levels of immune cell infiltration among the subtypes (Fig. 2C), suggesting that fatty acid-related genes likely influence the distribution of cells within the TIME, thereby leading to different sensitivities to immunotherapy and ultimately resulting in variations in individual prognosis. GSVA results (Fig. 2D) highlighted distinct activity levels in numerous immune, drug metabolism, and cancer-related pathways, including B/T/NK cell receptor signaling, toll-like receptor signaling, antigen processing and presentation, drug metabolism via cytochrome P450, and melanogenesis. GSEA outcomes also revealed pathway suppression or activation within the three subtypes (Fig. 2E). Notably, the antigen processing and presentation pathway in subtype A—the group with the most favorable outcomes—was significantly activated. Subtype B melanoma exhibited suppression of certain immune-related disease pathways. Conversely, subtype C exhibited an over-activation of the drug metabolism cytochrome P450 pathway, coinciding with the poorest outcomes.

### Construction fatty acid-related prognosis signature

**Fig. 3:**
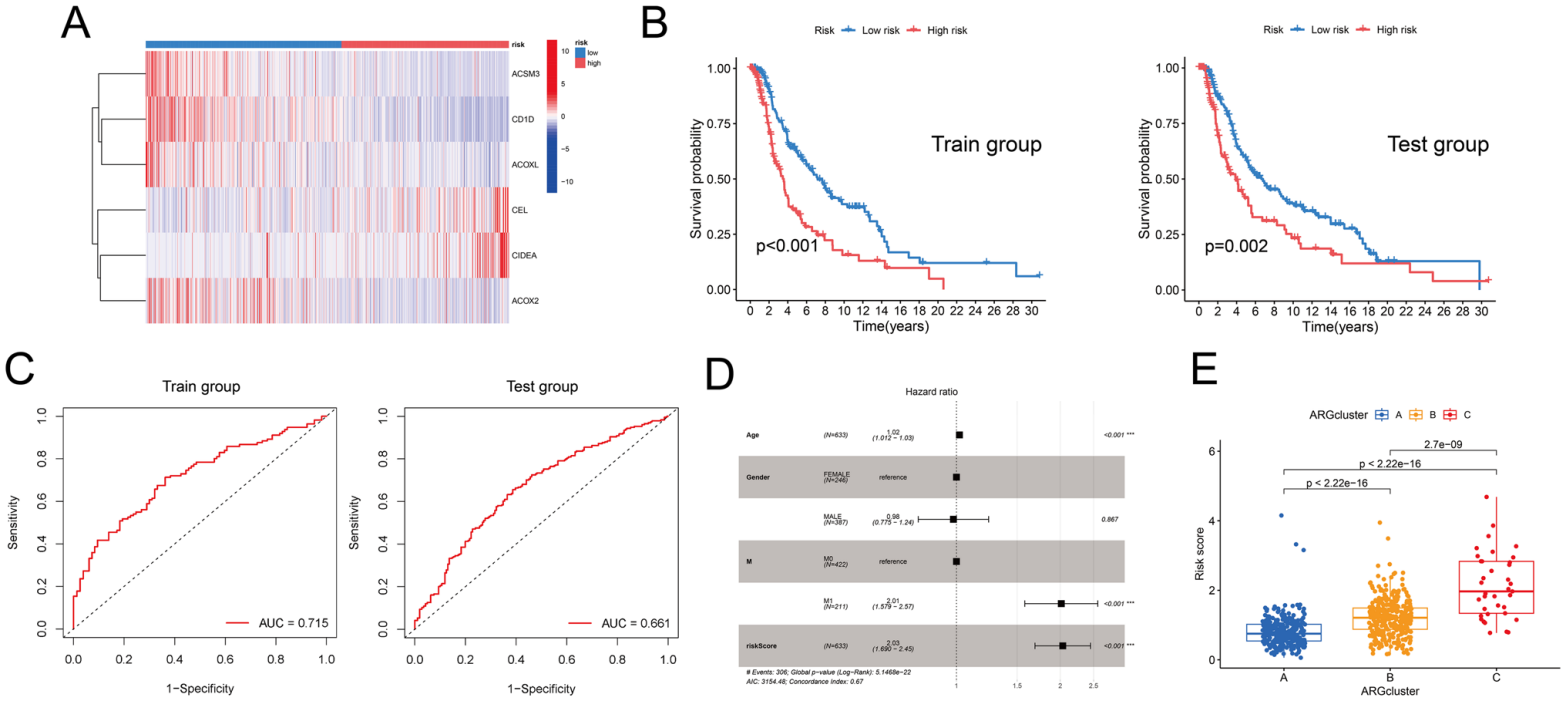
**A** heatmap displays the differences in the expression levels of the six genes between the high-risk and low-risk groups. **B** Survival analysis indicates that in both the train group and test group, patients with high-risk scores have significantly worse prognoses compared to those with low-risk scores. **C** The AUC values for the train group and test group are 0.715 and 0.661, respectively. **D** The risk scores were calculated for the three subtypes, and there were significant differences between each pair of subtypes. **E** Age, metastasis occurrence, and risk score can serve as independent risk factors for predicting patient prognosis

To examine the fatty-acid genes for the prognostic prediction of CM, 14 DEFAGs, which exhibited associations with overall survival in melanoma patients, underwent LASSO-Cox regression analysis. This analysis led to the establishment of a signature comprising six genes, as follows: ACSM3 expression * − 0.67595 + CD1D expression *  − 0.3307 + CEL expression * 0.52676 + CIDEA expression * 0.22805 + ACOXL expression * − 0.74305 + ACOX2 expression * − 0.24089. The median score, calculated from all samples, was used as the cut-off value to classify all cases into high- and low-risk groups. This classification was performed to facilitate subsequent inter-group analysis aimed at further evaluating the signature. Expression levels of these six candidate genes in high- and low-risk groups were visualized in a heatmap (Fig. 3A). Kaplan–Meier survival analysis demonstrated that melanoma patients with higher risk scores experienced worse prognosis in both the train and test sets (Fig. 3B). To evaluate the area under the ROC curve (AUC), a 5 years ROC curve was generated (Fig. 3C), yielding AUC values of 0.715 and 0.661 for the train and test sets, respectively, indicating that the signature exhibits good predictive capability. Subsequent multivariate Cox analysis established the risk score calculated by this signature as an independent prognostic risk factor for melanoma [Hazard ratio = 2.03, 95% CI (1.69, 2.45), *p* = 6.39e–14] (Fig. 3D). Furthermore, risk scores were computed for all samples in subtypes A, B, and C. Wilcox *t* test results indicated that subtype A exhibited the lowest risk scores, followed by subtype B, while subtype C showed the highest risk scores; these differences between groups were statistically significant (Fig. 3E). This reaffirms the influence of varied DEFAGs expression patterns on prognosis and supports the use of signature-derived risk scores as clinical prognostic factors.

### Establishment of a prognostic nomogram

**Fig. 4:**
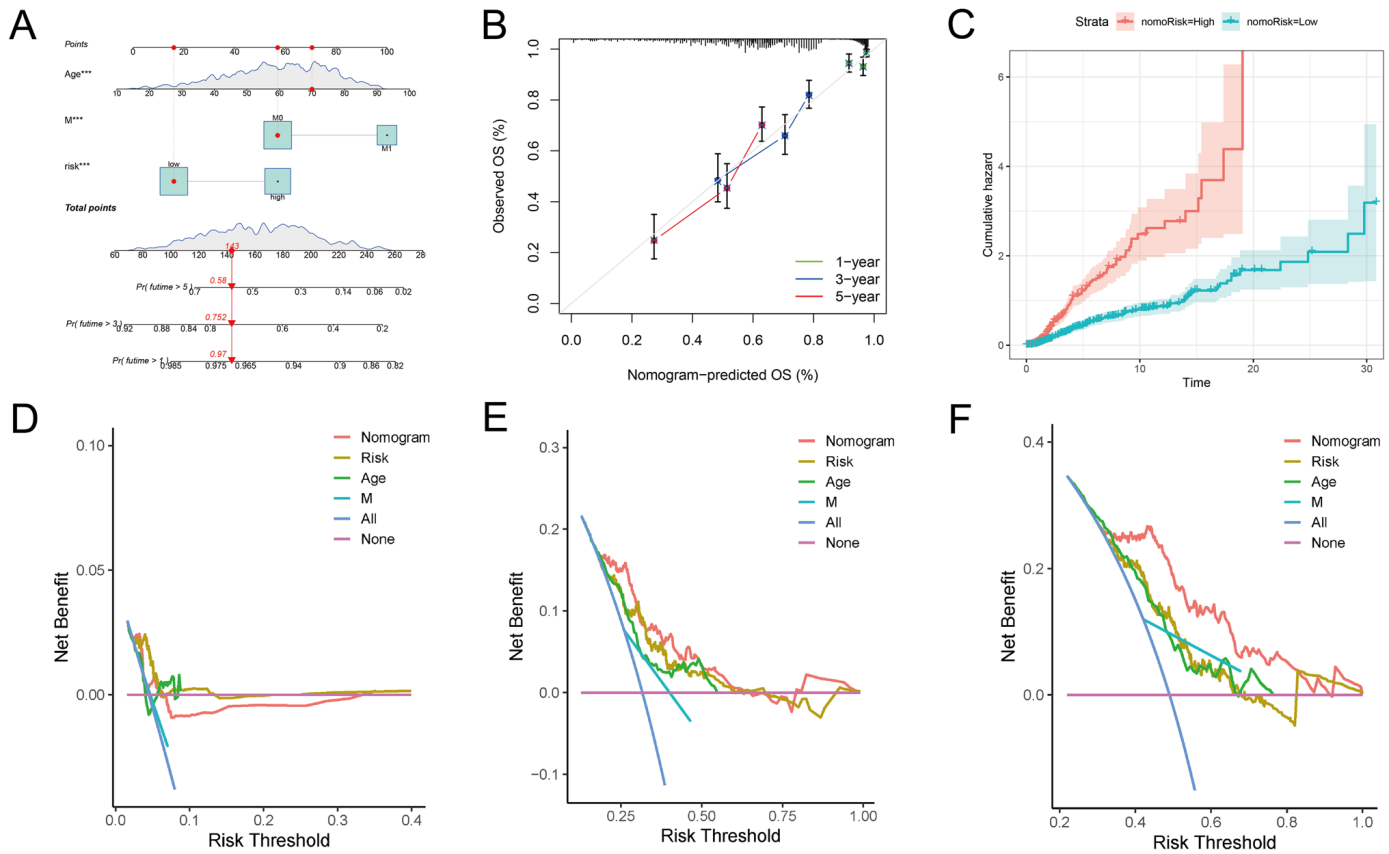
**A** Construction of a nomogram based on age, metastasis occurrence, and risk scores. **B** The calibration curve shows favorable alignment and actual observations for nomogram at 1, 3, and 5 years. **C** Patients with higher risk scores exhibited elevated cumulative hazard rates. **D**–**F** In the DCA, the nomogram model has improved prognostic accuracy at 3 and 5 years, but performs less favorably at 1 year

To enhance the prognostic capacity of the signature and facilitate its clinical feasibility, we further integrated independent clinical risk factors, including age and metastasis, into the prognostic signature to create a comprehensive nomogram for predicting the prognosis of melanoma patients within the TCGA-SCKM and GSE65904 cohorts at 1, 3, and 5 years. As shown in the nomogram, when patients were scored at 143, their 1 years, 3 years, and 5 years survival rates were 0.97, 0.752, and 0.58, respectively (Fig. 4A). A calibration curve was generated to assess the diagnostic performance of the nomogram model. The curves in the figure closely follow the diagonal line at 1, 3, and 5 years, indicating that the nomogram-based predictions of patient survival closely match the actual observed values, revealing a favorable alignment with real-world observations (Fig. 4B). The cumulative hazard analysis indicated that patients with higher risk scores exhibited correspondingly elevated cumulative hazard rates, providing a bidirectional validation of the nomogram and prognostic signature (Fig. 4C). In the DCA, the nomogram model outperformed age and metastasis alone, demonstrating improved prognostic accuracy at 3 and 5 years (Fig. 4D–F). Clearly, all risk factors, including the nomogram model, displayed limited predictive capability at 1 year, which is likely attributed to the relatively high 1-year survival rate among CM patients. Overall, the nomogram model demonstrates strong predictive capabilities for multi-year survival rates and outperforms the individual application of risk scores and other clinical risk factors.

### Immune microenvironment and immune response analysis

**Fig. 5:**
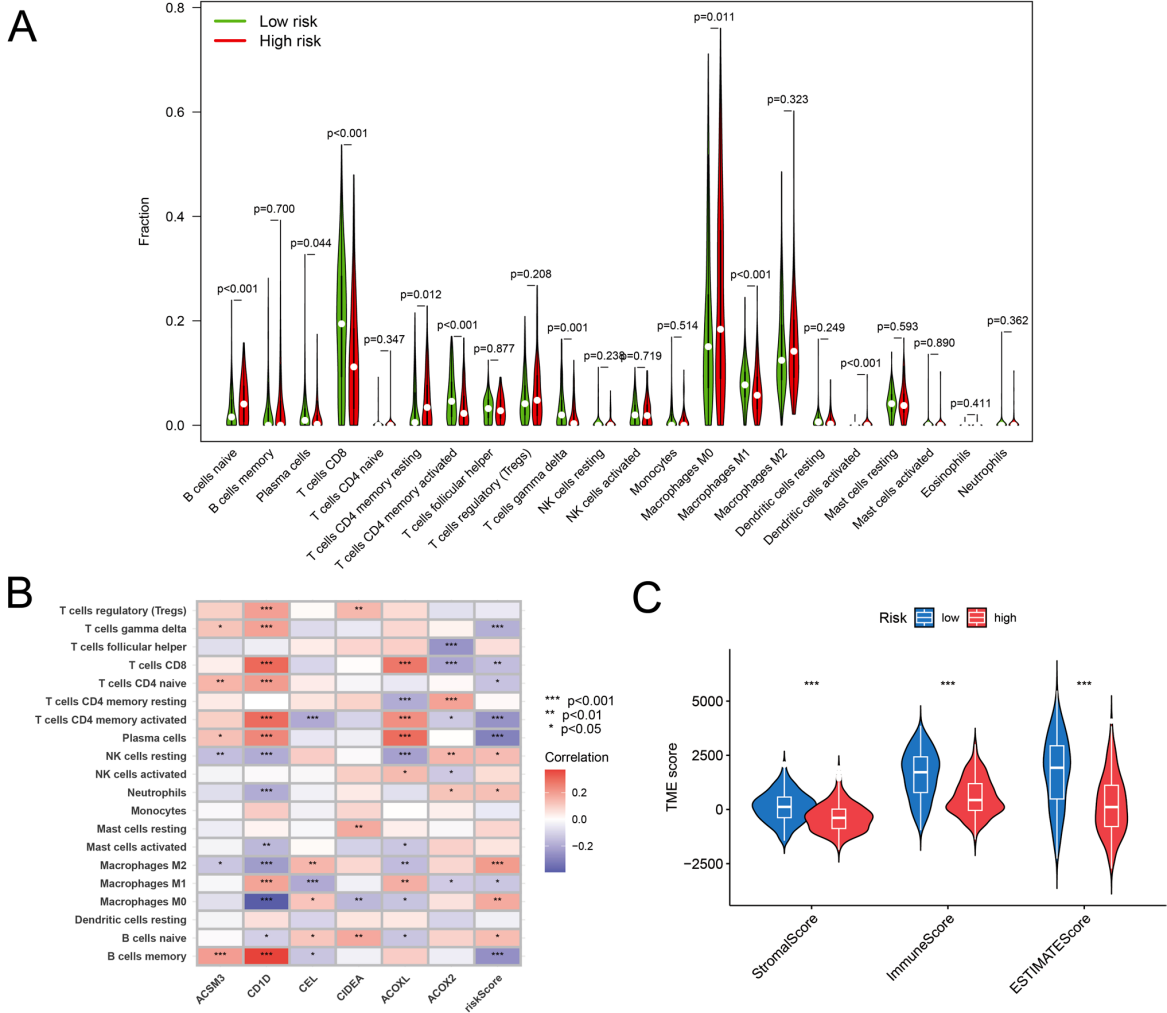
**A** Violin plot visualizes the differences in the proportions of 22 types of immune cells between the high- and low-risk groups. **B** Heatmap displays the impact of individual genes on the TIME. **C** Patients with high-risk scores exhibit lower StromalScore, ImmuneScore, and ESTIMATEScore (*p* < 0.001)

To investigate whether the signature has the ability to assist in identifying the immune cell composition within the TIME, we individually quantified the content of 22 different immune cell types in all samples and performed Wilcoxon tests. The results indicated that low-risk groups exhibited decreased infiltration levels of naïve B cells, memory B cells, and Macrophages M2 (*p* < 0.05); and elevated infiltration levels of plasma cells, activated memory CD4^+^ T cel ls, γδ cells, and M1 macrophages (Fig. 5A). Of course, some of these differences are attributed to the differential expression of DEFAGs. The impact of individual genes on the TIME is depicted in the heatmap (Fig. 5B). Notably, the expression of CD1D within the melanoma TIME influenced the infiltration of various immune cell types, particularly memory B cells, M0 macrophages, CD8^+^ T cells, and others. Furthermore, ACOXL also played a substantial role, affecting CD8^+^ T cells, plasma cells, activated/resting memory CD4^+^ T cells, and more. This suggests that CD1D and ACOXL have relatively prominent impacts on the TIME of CM patients, but it does not necessarily imply that these genes directly influence the distribution of specific immune cells. The underlying mechanisms may be quite complex. Detailed correlations between risk scores and the infiltration of 12 immune cell types are presented in Figure S1. Moreover, high-risk group were associated with lower stromal scores, immune scores, and ESTIMATE scores, indicating that the tumor purity is relatively low in the high-risk group (Fig. 5C), which is often closely linked to clinical issues such as poor immunotherapeutic responsiveness and unfavorable prognosis.

### Drug sensitivity analysis

**Fig. 6:**
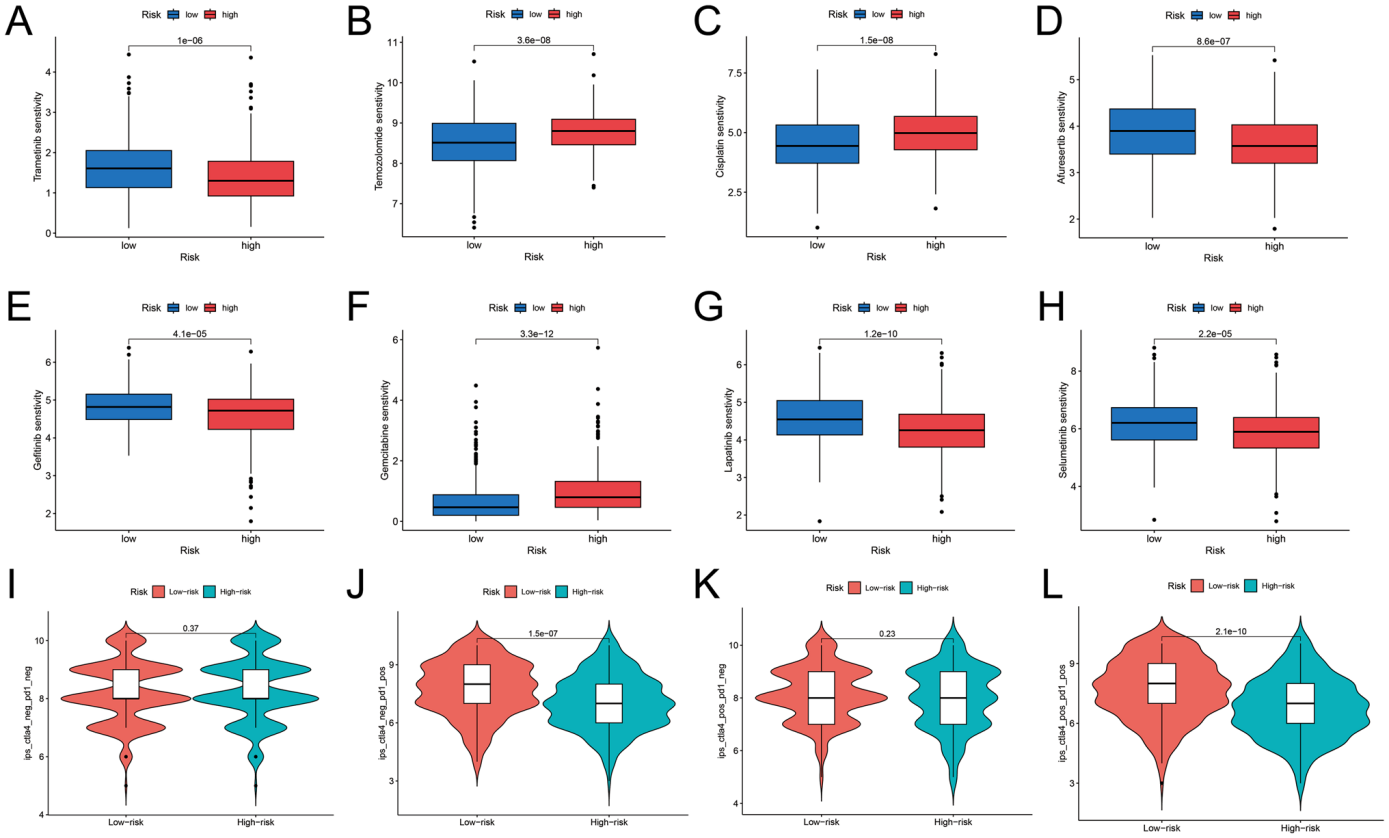
**A**–**H** Patients in low- and high-risk groups show different responsiveness to eight common drugs. **I**–**L** Among patients with PD1-positive expression, those in the low-risk group demonstrated superior immune therapy responses compared to their counterparts in the high-risk group

Using the oncoPredict R package, distinct responses to certain drugs were predicted between the low- and high-risk groups. Notably, trametinib, temozolomide, cisplatin, afuresertib, gefitinib, gemcitabine, lapatinib, and selumetinib displayed divergent responses (Fig. 6A–H). Additionally, the Immune Prognostic Score (IPS) was computed utilizing TCIA (The Cancer Immunome Atlas) data to predict immunotherapy sensitivity. Specifically, among patients with PD1-positive and CTLA4-negative or positive expressions, those in the low-risk group exhibited superior immune therapy responses compared to their counterparts in the high-risk group (*p* < 0.001). However, no statistically significant difference was observed among patients with PD1-negative expression between the two subgroups (*p* > 0.05) (Fig. 6I–L).

### CD1D was mainly expressed in monocytes and macrophages in TIME

**Fig. 7:**
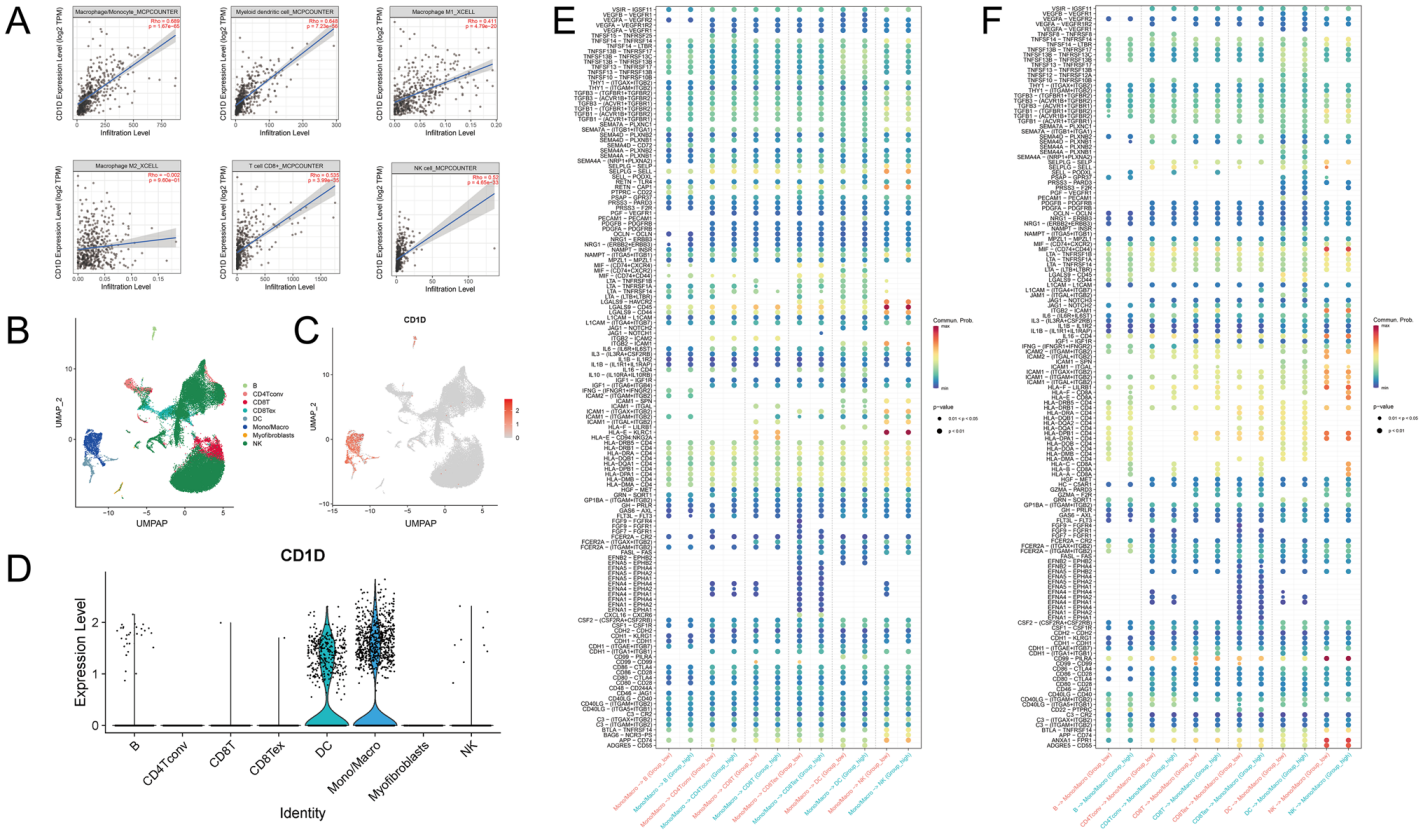
**A** The scatter plot visualizes the relationship between CD1D gene expression levels and the abundance of six types of immune cells. **B** UMAP plot displays the composition of eight main cell clusters derived from CM samples. **C** UMAP plot displays the expression level of CD1D in the whole cell clusters. **D** The violin plot visualizes the expression levels of CD1D in eight cell clusters. **E** The relationship between CD1D expression levels and signal transduction from monocytes/macrophages to other immune cells. **F** The relationship between CD1D expression levels and signal transduction from other immune cells to monocytes/macrophages

Among the six genes, CD1D was the most correlated gene with immune cell infiltration in the TIME. According to the Timer 2.0 database, CD1D expression was positively correlated with the infiltration levels of Macrophage/Monocyte, Dendritic cell, M1 macrophage, CD8^+^ T cell, and NK cell, but it showed no correlation with M2 macrophage infiltration (Fig. 7A). To further delve into the localization of CD1D within the TIME of cutaneous melanoma (CM) and its impact on the interactions between immune cells, we conducted an analysis of the single-cell expression profile in the GSE139249 dataset. Tumor samples were clustered and annotated into eight distinct clusters, identified as B cells, conventional CD4^+^ T cells, CD8^+^ T cells, exhausted CD8^+^ T cells, dendritic cells (DCs), monocytes or macrophages, myofibroblasts, and NK cells (Fig. 7B). CD1D was primarily expressed in dendritic cells (DCs), particularly in monocytes or macrophages (Fig. 7C–D). Furthermore, we conducted intercellular communication analysis to investigate the impact of CD1D expression levels on ligand–receptor signaling between monocytes/macrophages and other immune cells. The results indicated that there are no significant differences in signal transduction from monocytes/macrophages to other cells. However, certain signal transductions from other immune cells to monocytes/macrophages are significantly enhanced, particularly the (HLA-A/B/C/E/F)-CD8A signaling from NK cells to monocytes/macrophages (Fig. 7E–F).

## Discussion

CM is one of the most aggressive and metastatic cutaneous malignant tumors, with increasing morbidity and mortality worldwide, and is the 19th most common cancer (Holmes 2014). Early-stage CM is often overlooked by patients, while for those who develop metastasis, surgical and chemotherapeutic interventions yield unsatisfactory outcomes, resulting in a median survival time of less than 9 months. Targeted therapy and immunotherapy have made significant advancements, although inter-individual variances persist (Siegel et al. 2016). Hence, constructing a signature for predicting patient prognosis, clinical and immunological characteristics, as well as immunotherapeutic responsiveness, holds paramount significance. To facilitate rapid proliferation, cancer cells undergo metabolic network reprogramming to generate sufficient bioenergy, ensuring the sustenance of their essential biological functions (Kosti et al. 2020). Mitochondrial oxidative phosphorylation (mOxPhos) is recognized as a key contributor to tumor growth within cancer cells (Caro et al. 2012). Particularly under challenging circumstances like nutrient deficiency, lipid fatty acid oxidation (FAO) assumes a central role in supplying energy to cancer cells, thereby playing a significant part in the progression and potential metastasis of tumors (Kant et al. 2020; Rozeveld et al. 2020). For the treatment of CM, inhibiting its mitochondrial respiratory chain offers a potential strategy to overcome the inherent multidrug resistance, thus potentially forestalling or delaying therapeutic resistance. This approach is promising due to CM's heightened reliance on oxidative phosphorylation, setting it apart from many other types of cancers.

For the treatment of CM, inhibiting its mitochondrial respiratory chain can help overcome the inherent multidrug resistance of melanoma, thereby avoiding or delaying the development of treatment resistance. This is due to CM's heightened dependence on OXPHOS compared to the majority of other cancer types (Roesch et al. 2013). As a result, targeting the metabolic pathway of FA synthesis holds promise for CM treatment by disrupting the essential energy supply required for cancer cell proliferation (Vivas-Garcia et al. 2020). Up until now, almost all research endeavors have been confined to individual genes related to fatty acids, with comprehensive investigations into the relationship between fatty acids and malignant melanoma being notably scarce. Our research has established a robust predictive model for malignant melanoma, which holds the potential to facilitate patient prognosis prediction, offer insights into clinical and immunological characteristics, and predict immunotherapeutic responsiveness. Furthermore, our study may also pave the way for future in-depth inquiries, which could yield valuable insights.

The prognostic signature has six genes, each of which are associated with the prognosis of CM patients. ACSM3 (Acyl-CoA medium-chain synthetase-3) belongs to the family of fatty acid coenzyme A synthetases. It interacts with medium and long-chain FAs on the outer mitochondrial membrane to produce acyl-CoA, and participates in the processes of FAs synthesis, storage, and degradation (Costagli and Galli 1998). Research has reported that, based on the analysis of public datasets and validated through in vitro experiments, low expression of ACSM3 is correlated with a poorer prognosis in CM. ACSM3 expression levels show no distinct differences across different tumor stages, but its expression significantly increases in metastatic cases. Besides, its expression level is positively correlated with the infiltration of CD8^+^ T cells, macrophages, and dendritic cells within the TIME. Xenograft CM murine with ACSM3 overexpression show synergistic effects for BRAF inhibitors, without inducing additional toxicity (Zhu et al. 2020). In high-grade serous ovarian carcinoma, ACSM3 exerts its anti-tumor effects by suppressing the activation of AMPK, leading to a reduction in mitochondrial respiration and glycolysis (Yang et al. 2022). It similarly exhibits tumor-suppressive effects in other types of cancers, including hepatocellular carcinoma and acute myeloid leukemia (Ruan et al. 2017; Zheng et al. 2023). ACOX2 (Acyl-CoA Oxidase 2) belongs to peroxisomal acyl-CoA oxidases, it can convert very long-chain FAs to metabolites that can be targeted to the mitochondria through α- or β- oxidation (Svensson and Shaw 2016). While research on the involvement of ACOX2 in the onset of CM remains limited, relevant studies have been conducted in other types of cancer. ACOX2 expression is significantly reduced in the vast majority of non-small cell lung cancer (NSCLC) patients. Additionally, ACOX2 exhibits opposing effects on CD8^+^ T cell infiltration between LUAD and LUSC (Sui et al. 2022). A comprehensive study, integrating TCGA datasets, clinical samples, and both in vivo and in vitro experiments, suggests that ACOX2 impedes the progression of liver cancer through the PPARα pathway (Zhang et al. 2021). Besides, ACOX2 deficiency also presents in primary malignant cardiac tumors and estrogen receptor positive breast cancer (Zhou and Wang 2017; Bjorklund et al. 2015). For CEL (Carboxyl ester lipase), it was reported that it is highly expressed in breast cancer tissues and is associated with poor overall survival and clinical pathological characteristics (Cui et al. 2019). Lipids not only serve as a source of energy and constituents of cell membranes but also play a crucial role in regulating macrophage signal transduction and polarization, thereby exerting a central role in macrophage activation and function (Yan and Horng 2020). CD1D is a recognized lipid antigen-presenting molecule, and numerous studies have suggested its role in presenting glycolipid antigens to iNKT cells (Invariant natural killer T cells). Upon activation, iNKT cells secrete granzyme B and perforin, leading to the killing of target cells (Voskoboinik et al. 2015). In addition to the classical pathway, recent research indicates that CD1D serves as a critical regulator of lipid metabolism in macrophages, playing a key role in their immune function. CD1D regulates lipid uptake by controlling the internalization of the lipid transport protein CD36, thereby promoting macrophage activation, cytokine secretion, and polarization. Its deficiency results in macrophage metabolic reprogramming, altering macrophage phenotype and activation (Brailey et al. 2022). In our study, we found that CD1D is expressed in antigen-presenting cells and monocyte-derived macrophage clusters within the CM’s TIME, with minimal expression in other immune cells. We first provided the description of intercellular communication among immune cells in the CM's TIME under varying CD1D expression levels. CD1D expression levels primarily affect cell communication from other cell lineages to monocyte-derived macrophages, influencing the interaction between HLA-A/B/C/E/F and CD8A, while there is no significant difference in cell communication from monocyte-derived macrophages to other immune cells. CD8A encodes the CD8a chain of the CD8 protein, which plays a crucial role in cell-mediated immune responses and T cell development (Du et al. 2023). CD8A expression positively correlates with CD8^+^ T cell and M1 macrophage infiltration, and high-density CD8^+^ T cells in bladder cancer patients are associated with better immune therapy response and improved prognosis (Zheng et al. 2022; Jansen et al. 2019). HLA loci are categorized into two classes: HLA class I and HLA class II. HLA-A/B/C, part of class I, present antigenic peptides to CD8^+^ T cells by binding to T-cell receptors (TCRs). Meanwhile, HLA class II molecules present antigen peptides, crucial for identifying nonself, infected, or malignant cells. In cancer, tumor cells employ numerous mechanisms to evade immune surveillance, and the loss of HLA is one of these mechanisms (von Boehmer 1991).

Fatty acids’ role in cutaneous melanoma (CM) has gained recent attention. We've constructed a novel fatty acid-related CM prognostic model, predicting patient outcomes and drug sensitivity, while also identifying TIME characteristics. Our nomogram offers excellent 3–5 year outcome predictions. Among seven model genes, CD1D's function in the TIME is notable, supported by single-cell analysis using public data. However, this study does have limitations. This study relies solely on public data, possibly introducing selection bias. Future experiments and validation with larger clinical samples are of significant importance. Furthermore, CD1D's role in CM's TIME requires further research, integrating clinical data for improved accuracy.

## Conclusion

This study integrates transcriptomic data from public databases related to CM. It identifies six differentially expressed fatty acid-related genes with prognostic significance and uses them to construct a novel prognostic risk model with strong predictive capabilities. The risk score calculated by this model reflects to some extent the immune cell infiltration in the individual’s TIME and their sensitivity to drugs. The nomogram built based on this model exhibits robust prognostic predictive ability. The role of CD1D in the TIME of CM has been preliminarily analyzed at the single-cell level. The findings of this study will aid in risk stratification, prognostic assessment, and treatment response prediction for CM patients. Furthermore, this study provides insights and a theoretical foundation for future research on the role of fatty acid-related genes in CM.

### Supplementary Information

Below is the link to the electronic supplementary material.

Supplementary file1 (JPG 3663 KB) Correlations between risk scores and the infiltration of 12 immune cell types

Supplementary file2 (TXT 2 KB)

Supplementary file3 (XLS 8 KB)

## Data Availability

All data analyzed during this study are included in the Methods section and Supplementary files. For further inquiries or requests regarding the data, please contact the corresponding author on reasonable request.
