# Supplementary figures and images for: The fatty acid-related gene signature stratifies poor prognosis patients and characterizes TIME in cutaneous melanoma
Source: J Cancer Res Clin Oncol. 2024 Jan 27;150(2):40. doi: 10.1007/s00432-023-05580-7 (PMC10822006; doi:10.1007/s00432-023-05580-7)

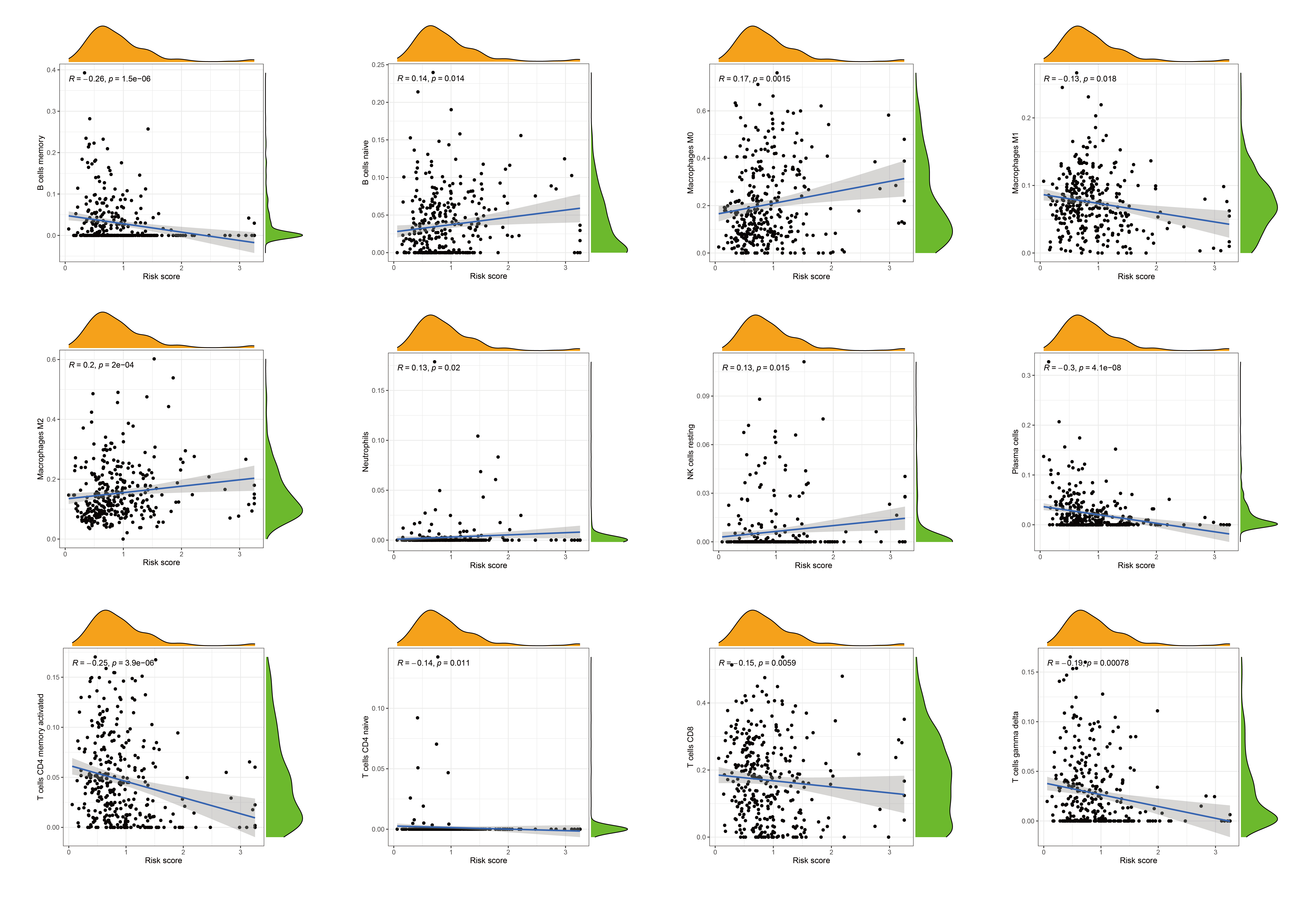

Supplement: Supplementary file 1 — Supplementary file1 (JPG 3663 KB) Correlations between risk scores and the infiltration of 12 immune cell types [file 432_2023_5580_MOESM1_ESM.jpg]
